# Combining *operando* synchrotron X-ray tomographic microscopy and scanning X-ray diffraction to study lithium ion batteries

**DOI:** 10.1038/srep27994

**Published:** 2016-06-21

**Authors:** Patrick Pietsch, Michael Hess, Wolfgang Ludwig, Jens Eller, Vanessa Wood

**Affiliations:** 1 Laboratory of Nanoelectronics, ETH Zurich, Switzerland; 2 European Synchrotron Radiation Facility (ESRF), Grenoble, France; 3 Mateis, INSA Lyon, UMR5510 CNRS, Villeurbanne, France

## Abstract

We present an *operando* study of a lithium ion battery combining scanning X-ray diffraction (SXRD) and synchrotron radiation X-ray tomographic microscopy (SRXTM) simultaneously for the first time. This combination of techniques facilitates the investigation of dynamic processes in lithium ion batteries containing amorphous and/or weakly attenuating active materials. While amorphous materials pose a challenge for diffraction techniques, weakly attenuating material systems pose a challenge for attenuation-contrast tomography. Furthermore, combining SXRD and SRXTM can be used to correlate processes occurring at the atomic level in the crystal lattices of the active materials with those at the scale of electrode microstructure. To demonstrate the benefits of this approach, we investigate a silicon powder electrode in lithium metal half-cell configuration. Combining SXRD and SRXTM, we are able to (i) quantify the dissolution of the metallic lithium electrode and the expansion of the silicon electrode, (ii) better understand the formation of the Li_15_Si_4_ phase, and (iii) non-invasively probe kinetic limitations within the silicon electrode. A simple model based on the 1D diffusion equation allows us to qualitatively understand the observed kinetics and demonstrates why high-capacity electrodes are more prone to inhomogeneous lithiation reactions.

Applications are demanding more from lithium ion batteries; most importantly higher energy density and faster kinetics. Charge- and discharge operation conditions strongly affect other battery performance parameters, such as effective specific charge and lifetime. Understanding the origins of limitations to lithiation dynamics at the material, electrode, and cell level is therefore crucial to developing next-generation batteries.

Synchrotron radiation provides the necessary time and energy resolution for non-invasive *operando* studies of dynamic processes occurring in lithium ion batteries during electrochemical operation. Recently, these X-ray synchrotron techniques have found widespread attention in the lithium ion battery research community^1^,^2^,^3^,^4^,^5^,^6^,^7^,^8^,^9^,^10^,^11^,^12^. While synchrotron X-ray diffraction techniques have been successfully applied to study the phase transformation mechanisms in LiFePO_4_ olivine structures^8^,^11^,^13^,^14^ for example, tomographic microscopy was shown to be an excellent tool to track volumetric changes and the resulting crack formation and mechanical degradation mechanisms in alloy anodes upon electrochemical cycling^4^. High-speed tomography and radiography were also used to image commercial 18650 cells during thermal runaway^2^.

Here, we present a combined synchrotron X-ray tomographic microscopy (SRXTM) and scanning X-ray diffraction (SXRD) experiment. By combining SRXTM and SXRD, we show that it is possible to obtain complementary information on the system simultaneously. The appearance and disappearance of crystalline phases and structural changes in them can be retrieved using SXRD, while volumetric and mechanical changes in the battery electrodes are obtained from the 3D tomographic measurements. Such complementary information is important for tracking lithiation dynamics in a battery where the anode and cathode contain different classes of active materials. Indeed, cathodes are predominately transition metal oxide intercalation compounds, while next-generation anodes will most likely contain alloying materials, such as silicon or tin integrated into a carbon matrix^15^,^16^.

To demonstrate the benefit of combining the two techniques, we perform SRXTM and SXRD measurements on a half-cell containing a thick silicon powder electrode that is potentiostatically lithiated at maximum load. We select silicon because, in addition to being a relevant material system^15^,^17^,^18^, upon lithiation, silicon undergoes phase changes from crystalline silicon through a variety of amorphous Li_x_Si_y_ compounds to crystalline Li_15_Si_4_^19^,^20^,^21^. Furthermore, these transformations result in a volumetric expansion of over 280%^22^,^23^ of the active material. We show that tomographic analysis enables us to quantify the volumetric changes of both electrodes, thereby tracking the dissolution of the lithium electrode and the expansion of the silicon electrode, while SXRD enables us to locally track the formation of the Li_15_S_4_ phase in the electrode. We use our findings from both measurements to build a model that provides insight into the kinetic limitations in anodes containing high capacity materials such as silicon.

## Results

### Setup and Data Analysis Approach

**Figure 1:**
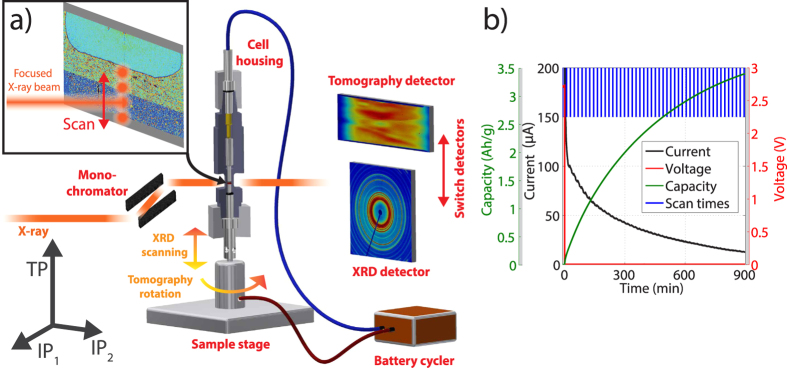
Experimental Setup (**a**) Sketch of the measurement setup for the combined operando scanning X-ray diffraction (SXRD) and synchrotron radiation X-ray tomographic microscopy (SRXTM) experiments. (**b**) Electrochemical characteristics of the measured sample; blue lines indicate the times of the imaging measurements.

Figure 1a shows a sketch of our measurement setup. The two techniques – SXRD and SXRTM - are continuously executed in alternating order, while the silicon electrode is potentiostatically lithiated at 10 mV.

For the SXRD measurements, the beam is focused on a 60 μm-sized spot in the middle of the cell using a set of compound refractive X-ray lenses, and the diffracted beam is collected by a detector in transmission geometry. During the measurement, the sample is shifted along the axis orthogonal to the current collector (i.e. the through-plane (TP) direction) (Fig. 1a inset) in order to capture depth-resolved information in the electrode. As indicated in the coordinate system in Fig. 1a, the other two axes parallel to the current collector are called the in-plane directions (IP_1_ and IP_2_).

For the SRXTM measurements, unfocused X-rays illuminate the entire sample volume, and the resulting projections are recorded by another higher resolution detector with 1.55 μm effective pixel size. Tomographic scans are performed by rotating the sample around its TP axis such that projections can be acquired every fraction of a degree (Fig. 1a).

Figure 1b summarizes the electrochemistry occurring in the cell during the SXRD and SRXTM measurements. The silicon powder electrode has an active mass of 175 μg. After a short, initial open-circuit period the potential is set to 10 mV and the current is recorded. The initial current reaches almost 1 mA (~1.6C), quickly drops to 100 μA (~C/6) within 20 min, and then slowly decays towards zero over the entire duration of the experiment. The vertical blue lines indicate the approximate times of the tomographic scans.

**Figure 2:**
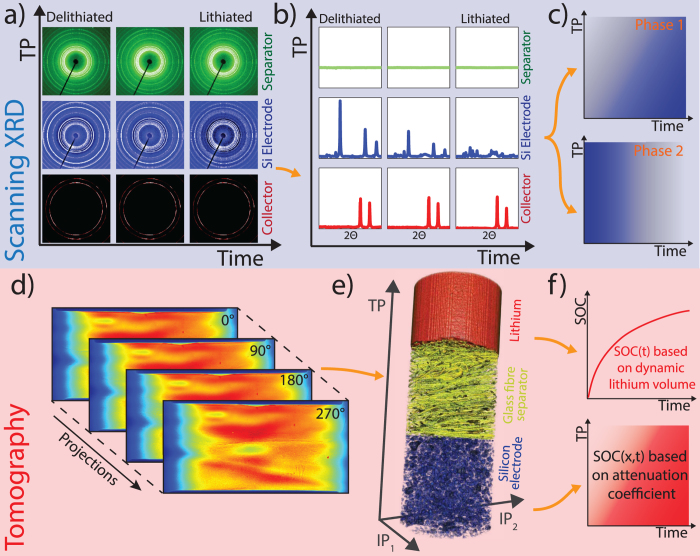
Data Processing Steps (**a**) Example of the 2D powder diffraction patterns from SXRD as a function of the TP space direction and time. (**b**) Processed 1D diffraction patterns at different levels in TP direction and different degrees of lithiation. (**c**) Sketch of phase maps that can be extracted from the SXRD patterns. (**d**) Example of acquired projections used for the tomographic reconstruction. (**e**) Rendering of a fraction of the sample after reconstruction. (**f**) Sketch of the information accessible from the tomographic measurements. The SOC is accessible from either the quantification of the lithium-metal dissolution (top) or from the change of X-ray attenuation within the silicon electrode (bottom).

Figure 2 shows the data processing approach for the SXRD and SRXTM measurements and the information that can be extracted from the data. In SXRD (Fig. 2a–c), the 2D diffraction patterns are collected as a function of time and depth in the electrode along the TP direction (Fig. 2a). All patterns are integrated and corrected for the background (Fig. 2b) using a series of post-processing steps described in the Supplementary Information. Integration of the respective XRD peak areas allows a semi-quantitative assessment of the amounts of the different phases that are present in the silicon electrode as a function of TP direction in the electrode and time (Fig. 2c).

In SRXTM (Fig. 2d–f), the projections are tomographically reconstructed to 3D volumetric datasets (Fig. 2d). In Fig. 2e, we show a rendering of a part of such a volume. With this data, we compute the dissolving lithium volume, which can be directly related to the amount of flowed electric charge using Faraday’s law (Fig. 2f, top). Furthermore, the time- and space-resolved state of charge (SOC) in the silicon electrode can be computed based on the change in X-ray attenuation resulting from the density change of silicon upon lithiation (Fig. 2f, bottom).

### Scanning X-ray Diffraction Analysis of the Silicon electrode

To shed light on the dynamics of formation and loss of crystalline phases in different parts of the silicon powder electrode, we investigate the results obtained from the SXRD measurements.

**Figure 3:**
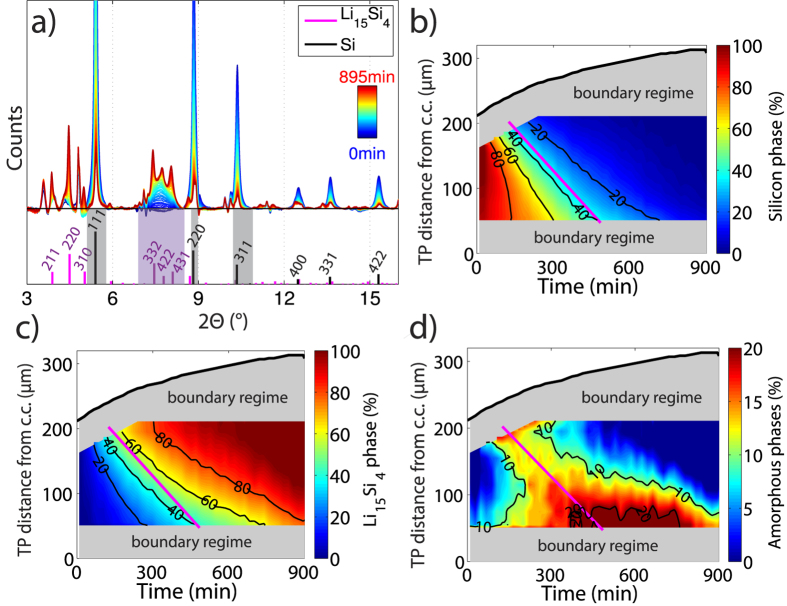
SXRD Measurements (**a**) Processed XRD patterns from the centre of the silicon electrode as a function of time. Reference peak positions for crystalline silicon (black lines) and Li_15_Si_4_ (magenta lines) based on data from^20^,^42^ are plotted below. (**b**) Normalized phase map representing the area under the three main XRD reflections of silicon as a function of TP direction and time (XRD peak area above the domains labelled with black shading in (**a**)). The magenta line represents the track of the lithiation front, while the black curve represents the upper edge of the expanding silicon electrode. Both lines are obtained from the tomographic information. (**c**) Equivalent map for the Li_15_Si_4_ phase (XRD peak area above the domains labelled with magenta shading in (**a**)). (**d**) Difference between 100% and the sum of the two maps in panels (**b**,**c**) as indicator for the amorphous phase. The data sets shown in (**b**–**d**) have been linearly interpolated to 4 *μm* spatial resolution for better visibility.

Figure 3a shows the processed diffraction patterns (Supplementary Information) at a fixed TP position in the middle of the electrode as a function of time. As the electrode is lithiated, peaks from the crystalline silicon phase vanish and a new phase gradually appears. Consistent with the findings from Obrovac and Christensen^19^, Chevrier, Zwanziger, and Dahn^20^, and Hatchard and Dahn^21^, we identify this phase as Li_15_Si_4_.

The amount of each phase in an illuminated volume can be quantified as a function of time and TP position within the cell by integrating the areas under the respective XRD peaks. To determine the amount of the silicon phase at a given depth in the electrode, we sum the areas under the {111}, {220}, and {311} peaks (black area). In analogy, to estimate the amount of the Li_15_Si_4_ phase, we consider the area under the {332}, {422}, {431}/{510} triple peak (magenta area). We transform the resulting integrated peak areas into phase fractions by adding an effective amorphous phase, representing intermediate amorphous Li_x_Si_y_ compounds, and applying the following normalization rules to the integrated peak areas:


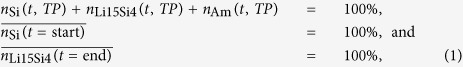


where the *n*_*i*_ with i = Si, Li_15_Si_4_, Am are the fractions of the corresponding phases, t is the time, and 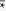 denotes the spatial average in the silicon electrode along the TP direction. With this approach, we can map the fractions of crystalline silicon, amorphous Li_x_Si_y_ compounds, and crystalline Li_15_Si_4_ as a function of the depth in the TP direction and time (Fig. 3b–d).

The black line in each map shows that the electrode thickness (i.e. the distance from the electrode-separator interface to the electrode-current collector interface (labelled as c.c.)) increases from 210 μm at t = 0 min to 310 μm at t = 900 min due to the expansion of the silicon. This information on electrode expansion is obtained from the tomographic data (see section ‘*Tomographic analysis of the silicon electrode’*).

**Figure 4:**
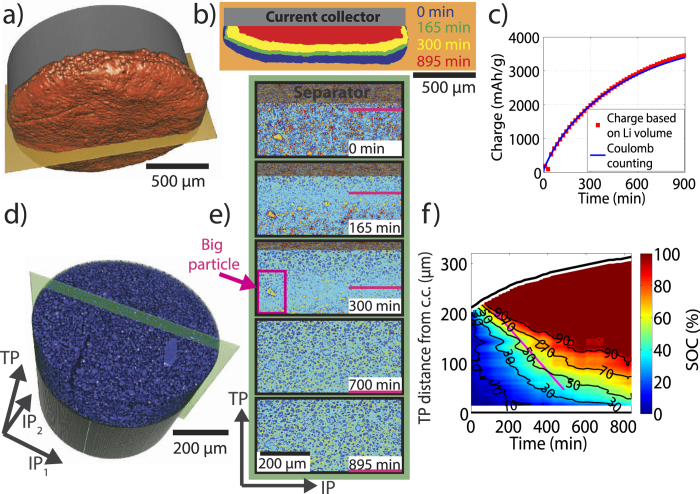
SRXTM Measurements (**a**) Rendering of the initial lithium counter-electrode. (**b**) Cuts through the lithium electrode along the orange plane at various times. (**c**) Comparison of the flowed charge as determined from (i) the battery cycler (blue) and (ii) the lithium electrode segmentation (red). (**d**) Rendering of a fraction of the silicon electrode. (**e**) Cuts through the silicon electrode along the green plane as a function of time. (**f**) SOC map based on the evaluation of the local attenuation coefficient in the silicon electrode. The magenta line represents the track of the lithiation front from (**e**), while the black curve represents the upper edge of the expanding silicon electrode.

The grey shaded regions in Fig. 3b–d indicate parts of the electrode that are excluded from the SXRD analysis. Close to the electrode separator interface, only a fraction of the photons penetrate the electrode at scanning positions due to a finite-size X-ray beam cross section. In regions into which the electrode expands, the absence of silicon at the initial time step in these domains makes normalization impossible. Close to the electrode current collector interface, we also cannot perform the XRD analysis. The XRD signal is disturbed by the presence of the steel current collector. Furthermore, the third normalization rule above assumes that silicon is fully converted to the Li_15_Si_4_ phase due to prolonged potentiostatic lithiation at 10 mV^19^,^21^; however, the measured flowed electric charge at the end of the experiment reaches only 95% of the theoretically possible value (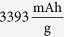 of 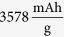). As shown in the Supplementary Information and Fig. 4, the 5% remaining specific charge is mostly located in the electrode region very close to the bottom current collector. We therefore limit our analysis of the SXRD data to the central region of the electrode (i.e., the area not greyed-out) where the silicon is fully converted to the Li_15_Si_4_ phase.

Figures 3b–d give insight into the lithiation dynamics occurring inside the silicon electrode. We find that the transformation from silicon to the Li_15_Si_4_ phase starts from the separator-electrode interface and then gradually progresses towards the current collector. The slopes of the contour lines in the silicon and the Li_15_Si_4_ maps reflect the velocities at which lithiation fronts (i.e. constant amounts of a given phase) move through the electrode. The magenta line maps the optically visible lithiation front, travelling at a velocity of 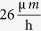, as obtained from analysis of the tomographic information (see section ‘*Tomographic analysis of the silicon electrode*’).

We also observe some nonlinear behaviour in the lithiation kinetics. Close to the separator, the decomposition of crystalline silicon is almost directly followed by the formation of the Li_15_Si_4_ phase, while, close to the current collector, the intermediate amorphous phases reside much longer until they slowly transform into the final Li_15_Si_4_ phase. In general, we can track inhomogeneous reactions where the silicon phase transitions are delayed with increasing distance from the Li-counter electrode. However, the amount of amorphous phase is below 20% throughout the experiment showing that these phases are only intermediates, which transform quickly to Li_15_Si_4_ when transport is not limited by diffusion through the porous electrode.

### Tomographic analysis of the lithium electrode

Turning our focus to the SRXTM analysis, we complement the results from the SXRD measurements by quantifying and visualizing the spatial progress of lithiation in both the silicon and the lithium electrode as a function of time. We start with the lithium electrode.

Using a sequence of image processing steps described in the Supplementary Information, we segment the metallic lithium electrode in each of the tomographic reconstructions. A rendering of the initial lithium electrode is depicted in Fig. 4a. In Fig. 4b, we show cuts through the lithium electrode along the orange plane as a function of time. As expected, we find that the lithium electrode shrinks considerably during the electrochemical reactions. Lithium dissolves rather homogenously on all surfaces, indicating a relatively uniform current distribution in the cell. A movie of the shrinking lithium electrode volume is available as Supplementary Information Video 2.

The volumetric lithium loss can be directly related to the accumulated charge via Faraday’s law:


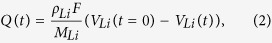


where *ρ*_*Li*_ is the mass density of metallic lithium, *F* is the Faraday constant and *M*_*Li*_ is the molar mass of lithium. Figure 4c shows excellent quantitative agreement between the charge calculated from the lithium volume and the charge obtained from integrating the current measured by the battery cycling system.

### Tomographic analysis of the silicon electrode

We next turn our focus to the porous silicon electrode depicted in Fig. 4d. In Fig. 4e, we show vertical cuts through the electrode along the green plane at various times after the onset of lithiation. While the full electrode is imaged at all times, the snap-shots in Fig. 4e show only a sub-window that is spatially fixed with respect to the laboratory frame.

At the beginning, t = 0 min, the glass fibre separator is visible at the top of the window (as labelled by the grey overlay), and the highly absorbing bright spots correspond to the pristine silicon particles in the electrode. Upon lithiation, the silicon particles expand by 280%^22^,^23^, which results in a gradual decrease in mass density and therefore a decrease in attenuation coefficient (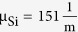 versus 
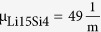
 as described in the Supplementary Information^20^,^24^). By time t = 895 min, the whole electrode is almost fully lithiated and the glass fibre separator is pushed out of the observation window.

The pristine silicon particles are more attenuating, while the fully lithiated silicon particles are less attenuating than the background (i.e. carbon black, binder and electrolyte). This leads to an inversion of contrast and a visible “lithiation front” as indicated by the magenta lines at the positions where the particle attenuation matches the effective attenuation of the background. The position of this front is calculated for each time step by selecting the electrode slice along the two IP directions that exhibits the smallest grey value standard deviation (see Supplementary Information). Starting from the electrode separator interface, this visible front moves downwards along the TP direction with an average velocity of 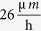 (see Supplementary Information).

Now, we generalize these findings by calculating the SOC distribution along the TP direction of the silicon electrode as a function of time. Because the local X-ray attenuation is related to the chemical composition and density of the active particles, their segmentation in each time step would allow for direct access of the local SOC^4^. However, the inversion of contrast prevents a consistent segmentation. As an alternative, we propose a simple model, discussed in the Supplementary Information, to determine the SOC as a function of time and the TP direction. It is based on the assumptions that the particles are distributed approximately homogeneously within the planes spanned by the IP directions and that the X-ray attenuation of the carbon black/binder/electrolyte background phase is constant over time. The results are summarized in the SOC map shown in Fig. 4f. Again, the top black line depicts the position of the separator-electrode interface along the TP direction as a function of time. Contour lines of constant SOC initially progress quickly towards the bottom current collector end eventually slow down.

Finally, SRXTM enables us to examine the active material, carbon black and binder volumetric fractions in the silicon electrode and their change upon lithiation. As a segmentation of the individual phases is not possible due to insufficient attenuation contrast, we compute the volumetric fractions based on our electrode composition by weight, the densities of silicon^20^, Li_15_Si_4_^20^, carbon (graphite)^25^ and PVDF binder^26^ and the overall electrode volume as determined from our tomographic measurements. We find that the volumetric fraction of the active material increases from 21% in the pristine electrode to 43% in the final state, while the pore volume fraction decreases from initially 54% to 44%. Due to the expansion of the electrode, also the volume fractions of the carbon black and the binder decrease from initially 11%, 14% to 6%, 7%, respectively, even though their absolute volume stays constant. This shows that the expansion of silicon upon lithiation is accommodated by both an increase of overall electrode volume (86%) and a slight decrease of the porosity. This porosity change suggests that very high pressures on the order of 1 GPa develop in the silicon electrode upon lithiation^27^, which is in agreement with our observation of crack formation within some silicon particles (Supplementary Information).

## Discussion

We now compare the results obtained from SXRD and SRXTM, and show that they are consistent, while also providing complementary information. Our findings from both techniques together present a global picture of the electrochemistry occurring in both the silicon and the lithium electrode. To understand the observed kinetic limitations in the silicon electrode, we introduce a simple model based on the 1D diffusion equation.

On one hand, SRXTM measurements allowed us to determine the local SOC distribution in the electrode based on the volumetric changes of the active particles or equivalently their X-ray attenuation coefficient^4^. SXRD can retrieve this information by tracking how the Li_15_Si_4_ phase forms while the silicon phase vanishes. While the data should be considered semi-quantitative due to the limitations discussed in the Supplementary Information, both techniques show good agreement as evident from a comparison of the Si and Li_15_Si_4_ phase fraction maps (Fig. 3b,c) with the SOC map in Fig. 4f obtained from SRXTM. In particular, the optically visible lithiation front extracted from the SRXTM measurements (see Fig. 4e and Supplementary Information) is approximately parallel to contour lines representing constant silicon or Li_15_Si_4_ phase fractions (Fig. 3b,c) that were obtained from the SXRD.

On the other hand, only SXRD allows us to detect and track the formation of the Li_15_Si_4_ phase, while only SRXTM provides the full 3D information necessary to analyse the volumetric changes of the two electrodes. In fact, SRXTM enables us to consider volume imbalance, loss, and expansion in the battery as a whole. While it is well known that the massive volumetric change of silicon upon (de)lithiation degrades the battery on the electrode level because of continuous electrolyte consumption in the solid electrolyte interface formation process and electric disconnection of active particles^17^, the volumetric changes of anode and cathode upon electrochemical cycling will also lead to volumetric changes on the cell or battery pack level. In our case, although silicon massively expands upon lithiation, we observe an overall volume loss of the battery sample as a whole, which results from the lithium volume loss overcompensating for the expansion of silicon (Supplementary Information Video 1). In fact, considering densities of 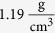 for Li_15_Si_4_^20^ and 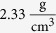 for silicon^20^, we find that the Li_15_Si_4_ phase exhibits volumetric charge capacities of 8335 Ah/(l silicon) or equivalently 2210 Ah/(l Li_15_Si_4_). This means that the volumetric increase of silicon upon full lithiation is 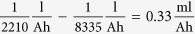. However, the volumetric loss of lithium is 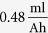, assuming a density of 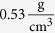
26, which overcompensates for the expansion of the silicon electrode, causing the overall battery volume to shrink during the course of lithiation. In fact, while from a gravimetric point of view, lithium is stored less efficiently in Li_15_Si_4_ compared to metallic lithium (1,857 Ah/(kg Li_15_Si_4_) versus 3,862 Ah/(kg Li)), this is surprisingly not the case when volumetric charge capacities are compared (2,210 Ah/(l Li_15_Si_4_) versus 2,062 Ah/(l lithium)).

Finally, we aim to use our combined SXRD and SRXTM study to understand the rate limiting effects occurring in our system. We observe that the silicon electrode lithiates first at the separator–electrode interface, and that the transformation of the amorphous Li_x_Si_y_ phases to the final Li_15_Si_4_ phase take place first in the top part of the electrode while the amorphous Li_x_Si_y_ phases reside much longer in the bottom part of the electrode (Fig. 3d). These findings imply that transport is limited by ionic diffusion rather than by electric conduction^28^,^29^.

We also note, however, that large silicon particles throughout the electrode continue to lithiate, even after the visible lithiation front has reached the bottom current collector (e.g. magenta box around the large particle in the cut at T = 300 min, Fig. 4e). This implies that the kinetics in the system are inhibited not only by the ionic transport in the electrolyte, but also by the solid state transport of lithium ions in the active material^30^.

To qualitatively understand the observed dynamic phenomena in the silicon electrode and their dependencies, we suggest a simple 1D diffusion-insertion model with our experimental parameters as input. We describe the concentrations of lithium ions in the electrolyte and in the silicon active material by *c*_*EL*_(*z, t*) and *c*_*Si*_(*z, t*), where *z* represents the TP distance from the bottom current collector and *t* denotes the time. Lithium ion transport through the electrolyte is governed by the 1D diffusion equation, with an additional empirical term

*k* *c*_*EL*_(*z, t*) describing the transfer of lithium ions from the electrolyte to the active material:


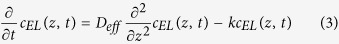



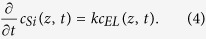



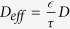
 is the effective diffusivity^31^,^32^ accounting for suppression of lithium ion transport by the electrode microstructure (i.e., finite porosity 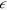 and tortuosity *τ*). We have assumed 
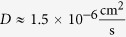
 as a typical diffusivity of lithium ions in carbonate electrolytes at room temperature^33^,^34^. The bottom boundary of the simulation domain is the electrode - current collector interface; the top boundary is the electrode - separator interface, which moves according to the experimental SRXTM data as shown in Figs 3b–d and 4f. At the top boundary, the influx of lithium ions is set to match the experimentally measured current by the battery charger. At the bottom boundary, no-flux boundary conditions are imposed. With the initial conditions set to *c*_*EL*_(*z*, *t* = 0) = *c*_*ini*_ and *c*_*Si*_(*z*, *t* = 0) = 0, the problem is uniquely defined. Further details on the model and a table with the input parameters used for the simulation can be found in the Supplementary Information.

**Figure 5:**
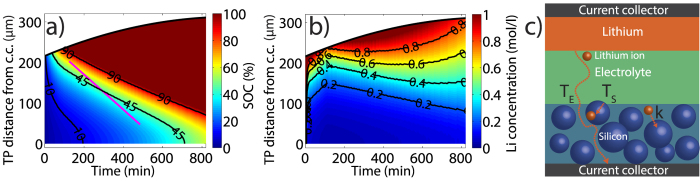
Simulation results (**a**) Simulation result for the SOC distribution *x*(*z*, *t*). The magenta line represents the track of the lithiation front from Figs 3b–d and 4e, while the black curve represents the upper edge of the expanding silicon electrode. (**b**) Corresponding concentration distribution in the electrolyte *c*_*E*_(*z*, *t*). (**c**) Sketch illustrating the time scale for lithium ion diffusion through the electrolyte *T*_*E*_, the time scale *Ts* on which a silicon particle lithiates and the rate constant *k*, describing lithium ion transfer from the electrolyte to the silicon particles.

We treat the only unknown parameters *k* and *τ* as variable input parameters and simulate the concentrations *c*_*EL*_(*z*, *t*) and *c*_*Si*_(*z*, *t*) in MATLAB. Normalizing the resulting concentration *c*_*Si*_(*z*, *t*) with the effective lithium concentration in a fully lithiated electrode *c*_*max*_(*t*) (see Supplementary Information) defines the SOC distribution, 
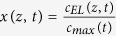
. Choosing *k* ≈ 30 h^−1^ and *τ* ≈ 2.5, we achieve qualitative agreement between the simulated distribution depicted in Fig. 5a and the experimental distributions in Figs 3b,c and 4f. The corresponding lithium concentration in the electrolyte *c*_*EL*_(*z*, *t*) is shown in Fig. 5b.

Because good agreement between experiment and simulation can be reached using reasonable values for the parameters *k* and *τ*, we conclude that the model contains the essential features needed to explain the SOC distribution. The major reason that the SOC gradients progress slowly through the electrode is the large specific charge capacity of silicon: only after the top parts of the electrode are almost fully lithiated, do subsequent lithium ions pass through to the bottom parts of the electrode, where lithiation reactions had previously stopped due to electrolyte depletion (see Fig. 5b). SOC gradients or inhomogeneities are hence much more likely to occur in high-capacity materials and can be mitigated only if the time scale for lithium diffusion in the electrolyte is orders of magnitude shorter than the time scale for lithium motion through the active particles.

To illustrate that in the case of our silicon electrode these time scales do not significantly differ, we use our model to compare order of magnitude estimates for the time *T*_*S*_ required to fully lithiate a silicon particle and the time *T*_*E*_ it takes a lithium ion to diffuse over a certain distance *δ* (see Fig. 5c). Approximating *c*_*EL*_(*z*, *t*) ≈ *c*_*ini*_, we have 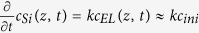 and thus 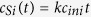. We require that *c*_*Si*_(*t*) = *c*_*max*_ at time *t* = *T*_*S*_, which yields:


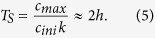


From a simple random walk model in one dimension^35^ we find


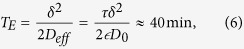


where we chose δ = 390 μm as the TP distance between the surface of the lithium electrode and the bottom current collector at t = 0 min based on our SRXTM data. We therefore conclude that *T*_*E*_ and *T*_*S*_ have similar orders of magnitude (hours) with *T*_*S*_ > *T*_*E*_. This means that both the reaction time of a silicon particle to fully lithiate and the time scale of diffusion through the electrolyte are comparable and in agreement with the experimental evidence.

In the Supplementary Information, we investigate the sensitivity of the model with respect to *τ*, *k*, and the specific charge capacity of the active material. We show, for example, that if the active material were graphite, with a specific charge capacity 10 times smaller than that of silicon, the electrode would be fully lithiated within only 25 min. We further emphasize that this simplified model only aims to capture the major kinetic effects occurring in the system.

Finally, our observations of (i) a porosity change within a pure silicon electrode^27^ and (ii) fracture of large silicon particles (Supplementary Information) suggest the presence of high stresses within the electrode such that stress-potential coupling effects^36^,^37^ are likely to participate in the lithiation dynamics. In particular, if stresses within the bottom part of the electrode were higher than in the top part, stress-potential coupling effects could contribute to the observed state of charge gradients along the TP direction of the electrode and the delayed formation of the Li_15_Si_4_ phase within the bottom part of the electrode.

## Conclusion

In conclusion, combined *operando* SXRTM and SXRD is a method to obtain a complete understanding of dynamic effects in lithium ion batteries. On the one hand, SXRD with a focused beam allowed us to (i) confirm the formation and map the dynamic distribution of the previously reported Li_15_Si_4_ phase and (ii) estimate the fraction of the intermediate Li_x_Si_y_ phases occurring in the system. On the other hand, 3D tomographic information allowed us to (i) quantify the dissolution of the lithium electrode due to the electrochemical reactions, (ii) characterize the SOC distribution throughout the silicon electrode, and (iii) identify solid state and electrolyte diffusion as rate limiting mechanisms in our system. Finally, a simple 1D diffusion transfer model reproduced the observed lithiation kinetics and highlighted why electrodes with a high specific charge capacity are more likely to suffer from inhomogeneous lithiation behaviour.

## Methods

### Electrochemical Cell

We construct an electrochemical cell housing (Fig. 1a) for *operando* X-ray measurements carried out in transmission geometry. The battery can consist of two arbitrary electrodes placed between two stainless steel current collectors. The upper current collector is connected to an electrically conductive spring-loaded probe pin, which accommodates for volumetric changes of the electrodes during operation and guarantees a well-defined pressure and good electrical contact throughout the experiment. The cell housing is made from polyether ether ketone (PEEK), an organic thermoplastic polymer with excellent mechanical and chemical properties.

### Sample preparation

The battery investigated here is a half-cell consisting of a 200 μm-thick silicon powder electrode with a diameter of 1.5 mm and an active mass of 175 μg, a 250 μm glass fibre separator (Whatman glass fibre filter), and a metallic lithium electrode (Alpha Aesar, lithium foil, 99.9%). The sample is wet with 30 μl standard LP50 electrolyte (1 M LiPF_6_ in 1 : 1 ethylene carbonate : diethyl carbonate, BASF) and assembled in the custom-made cell in an argon-filled glove box (O_2_ < 0.2 ppm). The silicon electrode is fabricated by combining amorphous/polycrystalline silicon powder (50 wt%, Alpha Aesar, 1–20 μm) and carbon black (25 wt%, Imerys, C65) with a pre-mixed solution containing 5 wt% PVDF (25 wt%, Kynar Flex^®^, HSV900) in NMP and agitated in a high shear mixer for 10 min. After ultra-sonication for 5 min and air bubble removal for 2 h, 200 μm thick films are coated on copper foil using a doctor blade. The resulting electrode sheets are dried in a vacuum oven at 120 °C for 8 h.

### Data acquisition

Measurements are carried out at the ID11 beamline at the European Synchrotron Radiation Facility (ESRF) in Grenoble using a beam energy of 42 keV. While examples of both *operando* tomographic imaging and *operando* X-ray diffraction studies have been carried out on lithium ion battery samples^4^,^11^, no studies to date apply both these techniques *operando*. The multi-purpose beamline ID11 at ESRF is suited for such combined experiments^38^,^39^. The measurement setup is depicted schematically in Fig. 1a. SRXTM and SXRD measurements are continuously performed in alternating order, while the silicon electrode is potentiostatically lithiated at 10 mV versus Li/Li+ (Fig. 1b).

For the tomographic scans, the XRD detector (Frelon 4 M) is moved out of the beam and a higher resolution detector system (50 μm thick X-ray luminescent screen, light optically coupled to a CCD camera using a 10x microscope resulting in 1.55 μm effective pixel size) is moved into the transmitted beam 10 mm away from the sample. The beam is defocused, illuminating the whole sample with a field of view of 1.59 mm × 0.93 mm. 1000 projections with 0.5 s integration time are acquired every fraction of a degree as the sample is rotated around its vertical axis by 360°. Collection of each set of projections takes 20 min.

For the XRD measurements, the higher resolution detector is moved out of the beam and the XRD detector is positioned in the transmitted beam 230 mm away from the sample. The beam is focused to a Gaussian profile with a FWHM of 60 μm using a set of compound refractive X-ray lenses. The sample is then scanned along the TP direction in steps of 20 μm to collect a powder diffraction scan series along the TP direction (Fig. 1a, inset). To achieve sufficient data quality we integrate the signal for 1s in each scanning position.

Over the course of the 15 h of lithiation, 48 tomographic scans (20 min each) and 48 SXRD scan series (1 min each) are continuously acquired (Fig. 1b). Switching detectors and automatic beam (de)focusing between the measurements take approximately 2 min.

### Processing diffraction data

From SXRD, we obtain 61, 2D patterns for each of the 48 time steps, resulting in about 3000 diffraction patterns. The geometrically measured sample-detector distance is slightly refined on a XRD pattern corresponding to the silicon electrode prior to lithiation using the software FIT2D^40^.

All patterns then undergo a sequence of post processing steps in MATLAB (see Supplementary Information). The cell housing material PEEK exhibits some crystallinity, which adds several additional Bragg reflections to the X-ray diffractograms. We remove this contribution by subtracting a background pattern in the electrolyte/separator domain from all patterns within a scan series.

### Tomographic data reconstruction

From the tomographic projections, the attenuation coefficients are reconstructed with standard filtered back-projection algorithms^41^ to yield a time series of 48 volumetric datasets, each with a size of 1,024 × 1,024 × 600 voxels in 32 bit floating point representation. The voxels have a size of 1.55 μm × 1.55 μm × 1.55 μm.

### Simulation

The simulation has been performed on a rectangular grid with spacing *dz* = 2 μm and time increment *dt* = 0.2 s using an in-house code written in the software MATLAB.

## Additional Information

**How to cite this article**: Pietsch, P. *et al*. Combining *operando* synchrotron X-ray tomographic microscopy and scanning X-ray diffraction to study lithium ion batteries. *Sci. Rep.* **6**, 27994; doi: 10.1038/srep27994 (2016).

## Supplementary Material

Supplementary Information

Supplementary Video 1

Supplementary Video 2
