# Supplementary material for: Combining operando synchrotron X-ray tomographic microscopy and scanning X-ray diffraction to study lithium ion batteries
Source: Sci Rep. 2016 Jun 21;6:27994. doi: 10.1038/srep27994 (PMC4914858; doi:10.1038/srep27994)
Supplement: Supplementary Information [file srep27994-s1.pdf]

Supplementary Information for

Combining *operando* synchrotron X-ray tomographic microscopy and scanning X-ray diffraction to study lithium ion batteries

Patrick Pietsch[1], Michael Hess[1], Wolfgang Ludwig[2,3], Jens Eller[1] and Vanessa Wood[1]

[1] Laboratory of Nanoelectronics, ETH Zurich, Switzerland

[2] European Synchrotron Radiation Facility (ESRF), Grenoble, France

[3] Mateis, INSA Lyon, UMR5510 CNRS, Villeurbanne, France

Overall cell volume change

To demonstrate the volumetric changes in the silicon and the lithium electrodes, we show a vertical cut of the full scans in the initial delithiated state (**Figure S1 a**) and the final lithiated state (**Figure S1 b**). The total volume of the battery slightly decreases as evident by the positions of the current collectors.

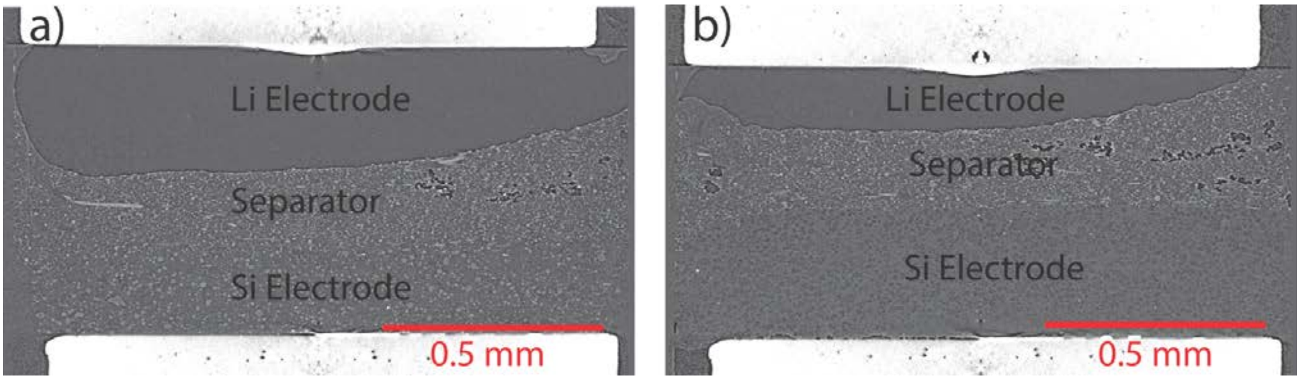

Figure S1 | Cell volume change (**a**) Cut through the sample in the initial stage. (**b**) Cut through the sample at the end of the lithiation process.

A video (**Supplemental Video 1**) showing the processes in the battery from the above perspective during all time steps is available.

Remaining delithiated domains in the electrode at the end of the experiment

Figure S2 shows a slice through the tomographic data of the silicon electrode at a TP position very close to the current collector at the very end of the experiment. A domain containing pristine or at least not fully lithiated silicon particles is visible, suggesting that the remaining 5 % of the theoretical capacity arises mostly from these domains in the very bottom part of the electrode. No such domains are found within the central and top parts of the electrode.

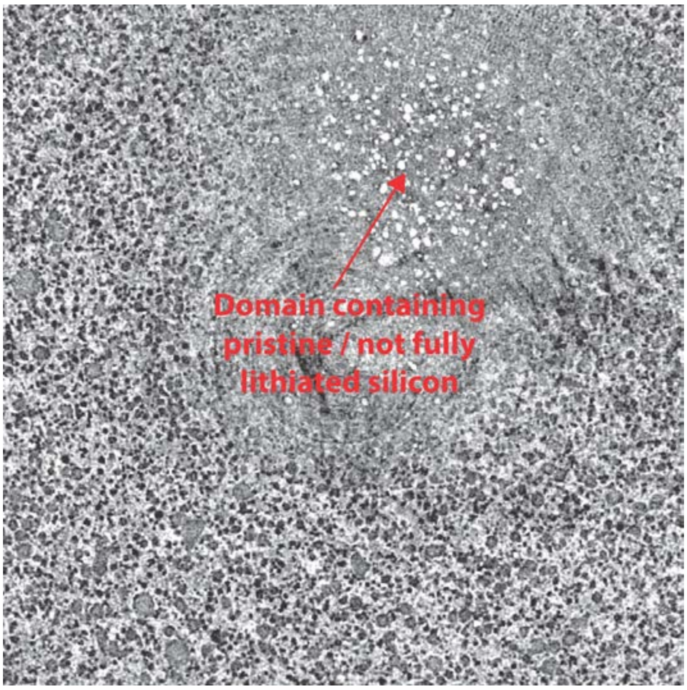

Figure S2 | Remaining delithiated domains in the electrode at the end of the experiment. Slice through the tomographic data of silicon electrode at a TP position very close to the bottom current collector at the very end of the experiment.

Image processing steps to isolate and analyse the lithium electrode

To segment and separate the lithium electrode, a sequence of image processing steps was applied to the tomographic data using the software MATLAB. These processing steps were applied to the full 3D datasets, but here, for ease of depiction, we show the effects of the processing steps on a single slice (**Figure S3**). As lithium is very weakly attenuating X-rays, we exploited edge enhancement effects at the lithium-separator interfaces to segment the electrode. All 48 tomographic scans at different time steps were processed in the same way with the exact same set of parameters.

1. Detection of the lithium - current collector interface identified by the large change in attenuation (highly attenuating steel versus weakly attenuating lithium). This interface defines the top of the segmented lithium volume.
2. Noise reduction with a wiener filter using a 3x3 neighbourhood (wiener2)
3. Data binarization using the triangle threshold method (custom function based on Zack, G. W., Rogers, W. E. & Latt, S. A. Automatic measurement of sister chromatid exchange frequency. *J. Histochem. Cytochem.* **25,** 741–753 (1977))
4. Removing connected background domains with less than 4 voxels (bwareaopen)
5. Dilation of the background contours with a ball structuring element of radius 2 voxels (imdilate)
6. Removing connected background domains with less than 400 voxels (bwareaopen)
7. Dilation of the background contours with a ball structuring element of radius 2 voxels (imdilate)
8. Removing connected background domains with less than 8000 voxels (bwareaopen)
9. Dilation of the background contours with a ball structuring element of radius 4 voxels (imdilate)

10. Pick the largest connected foreground domain (bwconncomp)
11. Erode background domains with a ball structuring element of radius 2+2+4=8 voxels (imerode)
12. Remove remaining ring artefacts (custom function from E.M. Lauridsen, G. Johnson & P. Tafforeaux, available at ID19, ESRF)
13. Remove remaining isolated background domains (imfill)

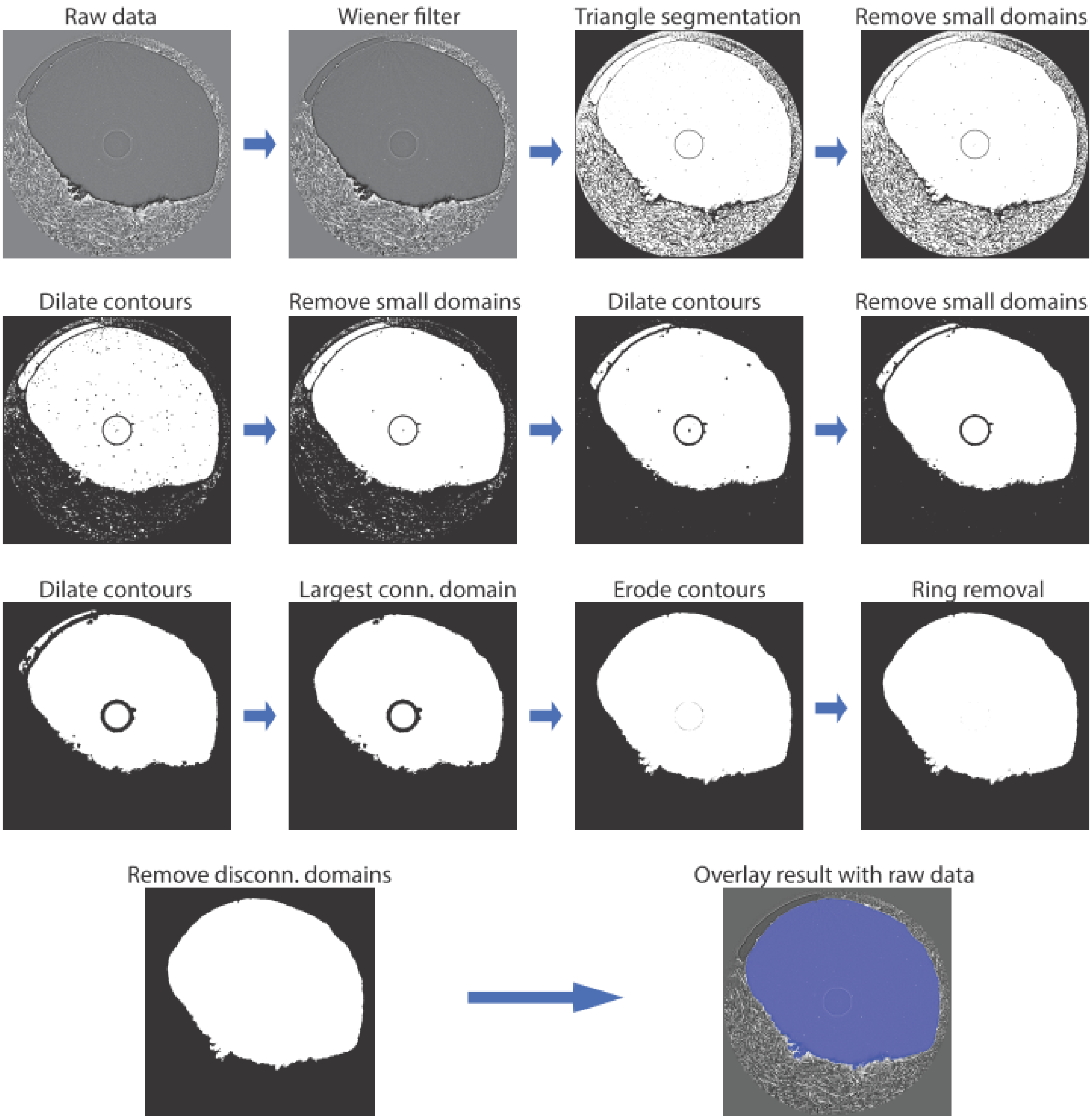

Figure S3 | Image processing steps to isolate and analyse the lithium electrode

A video (**Supplemental Video 2**) showing a 3D rendering of the electrode as a function of time highlights the shrinking of the lithium electrode.

Determination of the lithiation front dynamics based on inversion of the attenuation contrast

600x600x260 voxel^3 sub-volumes were cropped out from the same location in each dataset. The location was chosen such that it (i) contained the complete silicon electrode in TP at all times and (ii) contained as much of the electrode in the IP directions as possible without including parts belonging to the cell housing.

For each slice the standard deviation in space of the grey value in the 1-norm was computed as a function of time, as depicted in **Figure S4 a**.

The minima correspond to the times when the lithiation front reaches the respective slices. Within the central regime of the electrode that is neither too close to the interface with the separator nor to close to the interface with the current collector, the resulting relation reflects an average velocity of 26 $\frac{\mu m}{h}$ (**Figure S4 b**).

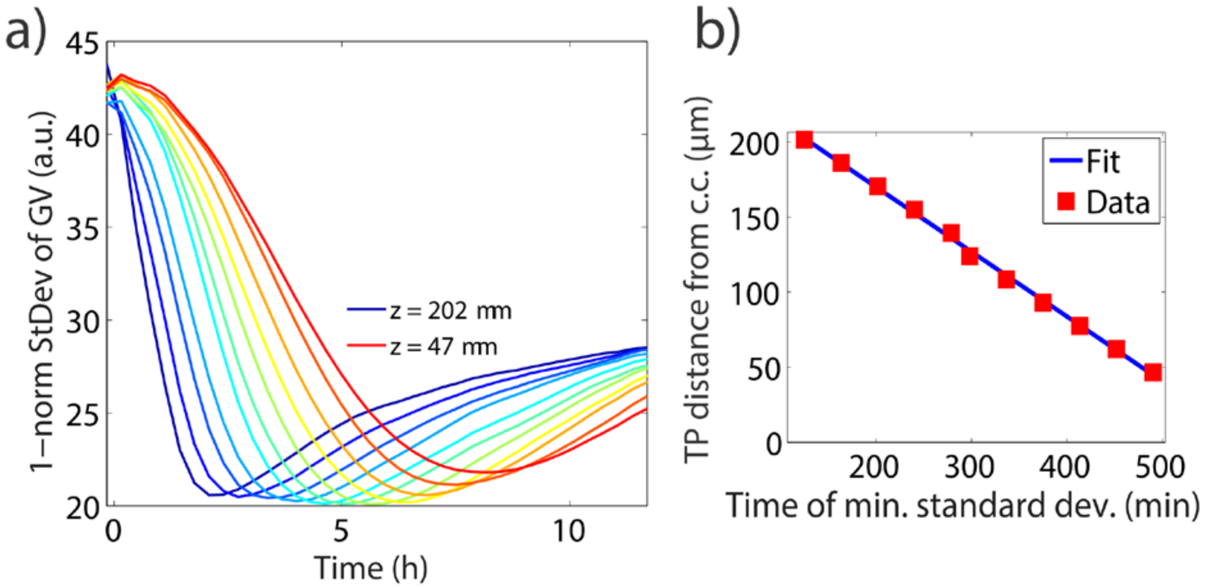

Figure S4 | Determination of the lithiation front dynamics based on inversion of the attenuation contrast (**a**) 1-norm standard deviations of the silicon electrode grey value as a function of the electrode slice number (TP direction) and time. (**b**) Position of the minima as a function of time

State of charge model

Figure S5 a shows the theoretical changes in density and attenuation of the various lithium-silicon compounds at 42keV calculated based on the mass attenuation coefficient tables of the NIST database (Hubbell, J. H. & Seltzer, S. M. Tables of X-Ray Mass Attenuation Coefficients and Mass Energy-Absorption Coefficients from 1 keV to 20 MeV for Elements Z = 1 to 92 and 48 Additional Substances of Dosimetric Interest*. (1995)) and crystallographic data of the various Li_xSi_y phases taken from Chevrier, Zwanziger and Dahn (Chevrier, V. L., Zwanziger, J. W. & Dahn, J. R. First principles study of Li–Si crystalline phases: Charge transfer, electronic structure, and lattice vibrations. *J. Alloys Compd.* **496,** 25–36 (2010)).

We quantified the state of charge, x, in the silicon powder electrode as a function of time, t and the depth, z, along the TP direction, where the position of the current collector is set to $z = 0$. We used the following simple model: for each slice (corresponding to the two IP directions) within the silicon powder electrode, the 2D spatial average X-ray attenuation coefficient $\bar{\mu}(x,z)$ will be given by:

$$\bar{\mu}(x,z) = p(x)\mu_a(x,z) + \big(1 - p(x)\big)\mu_b \qquad \textit{Equation 1}$$

where $p(x)$ describes the volumetric fraction of the active material in the electrode, $\mu_a(x,z)$ describes the local attenuation coefficient of the electrochemically active material and μ_b describes the effective attenuation coefficient of the inactive background material, which is meant to lump together attenuation properties of the electrolyte, binder, and carbon black.

Based on the observation that the active material expands approximately linearly as a function of its lithium loading (**Figure S5 a**), we assume that $p(x)$ is also linear in x:

$$p(x) = (p_1 - p_0)x + p_0 \qquad \textit{Equation 2}$$

Here, p_1 and p_0 describe the active material volumetric phase fractions at the experimentally determined states of charge at the beginning (0%) and the end (95%) of the experiment. They can be estimated from the active mass, the densities of the silicon phase and the $Li_{15}Si_4$ phase and the electrode volume in the initial and the final time steps as explained in the main text.

$\mu_a(x)$ can be approximated by a second order polynomial fit to the theoretical data shown in **Figure S5 a**

$$\mu_a(x) = ax^2 + bx + c \qquad \textit{Equation 3}$$

Equation 2 and Equation 3 can be inserted into Equation 1. The resulting equation can be solved for the state of charge as a function of the experimentally accessible quantity $\bar{\mu}(z,t)$ and the parameters p_0, p_1, a, b, c, and μ_b. a, b, and c are obtained from the second order least squares fit. μ_b is estimated from a comparison of the grey value histograms over the entire electrode at the initial and the final time steps.

To compensate for X-ray intensity variations over time and for slightly non-uniform particle distribution along the electrode z-direction, we normalize the average grey value of each slice in each time step by the corresponding grey value of the current collector and the grey value profile at time step 0:

$$GV_{corr}(z,t) = \frac{GV(z,t)}{GV_{c.c.}(t)GV(z,t=0)} \qquad \textit{Equation 4}$$

We then slightly smooth the data and linearly remap it in such a way that the grey value at the initial time step corresponds to $\bar{\mu}(x=0)$ and the grey value at the final time step corresponds to $\bar{\mu}(x=0.95)$. Finally, we use our equation $x(\bar{\mu}(z,t);\ p_0, p_1, a, b, c, \mu_b)$ (**Figure S5 b**) to approximate the state of charge in the electrode as a function of depth z and time t.

It should be mentioned that this model for the dynamic lithiation process in the silicon electrode exhibits several limitations, such as the assumptions made in the model and the fact that local tomography (the cell housing does not completely fit into the field of view) is intrinsically not quantitative. Also, as the intermediate Li_xSi_y phases (**Figure S5 a**) have not been observed to crystallize upon electrochemical operation at room temperature (Obrovac, M. N. & Christensen,

L. Structural Changes in Silicon Anodes during Lithium Insertion/Extraction. *Electrochem. Solid-State Lett.* **7,** A93–A96 (2004) and Hatchard, T. D. & Dahn, J. R. In Situ XRD and Electrochemical Study of the Reaction of Lithium with Amorphous Silicon. *J. Electrochem. Soc.* **151,** A838–A842 (2004)), deviations from the theoretical state of charge – attenuation coefficient characteristic as calculated in **Figure S5 a** might occur. The model should therefore only be considered semi-quantitative, but nevertheless delivers valuable insights.

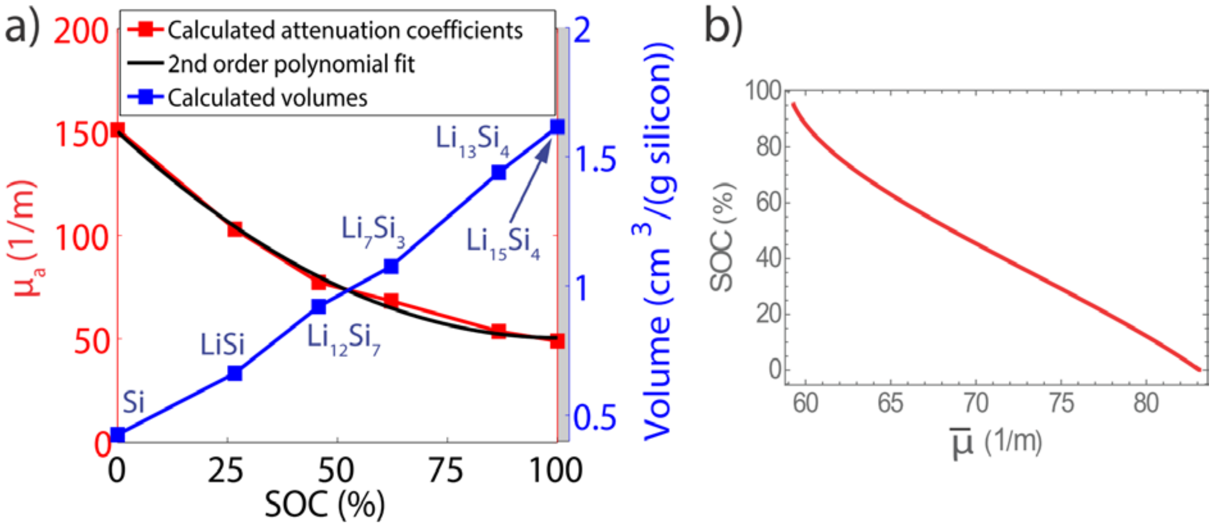

Figure S5 | State of charge model (**a**) Volume and attenuation coefficient of the Li_xSi_y phases as a function of the state of charge. (**b**) State of charge - $\bar{\mu}$ relation resulting from the model.

Processing of XRD patterns

After refinement of the sample detector distance based on an initial diffraction measurement in the silicon electrode (software FIT2D from ESRF), the approximately 3000 2D X-ray diffraction powder patterns all underwent the same chain of processing steps using the software MATLAB. All parameters were identical for all scanning positions and time steps:

1.) Correction of spatial detector non-linearity on each pattern using calibration files from ESRF ID11 beamline.
2.) Determination of the exact centre of rotation of the patterns for each spatial SXRD sequence
3.) Angular integration of all patterns to obtain 1D powder diffraction patterns
4.) Subtraction of the background using spline interpolation
5.) From each pattern in a SXRD series we subtracted one pattern taken at the position of the electrolyte. This step compensates the contribution of the partially crystalline PEEK cell housing to the XRD patterns. Remaining artifacts of this step can be recognized in the patterns at low diffraction angles (see main text **Figure 3a**).
6.) Correction for non-constant incoming X-ray flux (slow linear decay between synchrotron ring electron refills)

The resulting data was spatially correlated to the tomographic data using the signals of the steel current collectors at the top and at the bottom.

Simulation of lithiation dynamics

The concentration of lithium ions $c_{EL}(z,t)$ traveling in the electrolyte through the silicon electrode along the TP coordinate z is defined by the 1D diffusion equation

$$\frac{\partial}{\partial t} c_{EL}(z,t) = D_{eff} \frac{\partial^2}{\partial z^2} c_{EL}(z,t) - k c_{EL}(z,t),$$

where $D_{eff} = \frac{\epsilon}{\tau} D$ is the effective diffusion constant with the porosity ϵ, the tortuosity τ and the diffusion constant in pure electrolyte D. We describe the exchange of lithium ions from the electrolyte to the active silicon material by the additional empirical transfer term $k \cdot c_{EL}(z,t)$. The effective concentration of lithium ions in the silicon active material $c_{Si}(z,t)$ is hence defined by

$$\frac{\partial}{\partial t} c_{Si}(z,t) = k c_{EL}(z,t).$$

The exchange constant k is locally set to zero when silicon particles are fully lithiated.

Due to the expansion of the electrode, the electrode - separator interface defines the position of a moving boundary $z = d(t)$, where lithium ions enter the electrode domain at a rate defined by the measured electric current $I(t)$:

$$\frac{I(t)}{FA} = J_{z=d(t)}(t) = -D_{eff} \frac{\partial c_{EL}(z,t)}{\partial z}\bigg|_{z=d(t)}$$

Here F is the Faraday constant, A is the geometric surface area of the electrode, and $J_{z=d(t)}(t)$ is the lithium ion flux at the moving boundary. In analogy there is a zero flux boundary condition at the electrode - current collector interface defined by $z = 0$:

$$0 = J_{z=0}(t) = -D_{eff} \frac{\partial c_{EL}(z,t)}{\partial z}\bigg|_{z=0}$$

In the beginning of the experiment, lithium ions exist in the electrolyte at a concentration c_{ini} and the silicon electrode is fully delithiated

$$c_{ini} = c_{EL}(z,t)|_{t=0}$$

$$0 = c_{Si}(z,t)|_{t=0}$$

The resulting system of equations is discretized on a grid with a spatial resolution dz and a time resolution dt and solved in MATLAB.

The resulting concentration $c_{Si}(z,t)$ defines the local state of charge distribution:

$$x(z,t) := \frac{c_{Si}(z,t)}{c_{max}(t)} := c_{Si}(z,t) \frac{FAd(t)}{m_A C_{Si}}$$

where $c_{max}(t) = \frac{m_A C_{Si}}{FAd(t)}$ is the effective lithium concentration at which silicon is fully lithiated and $d(t)$ is the time-dependent electrode thickness. m_A and C_{Si} represent the active mass in the electrode and the specific gravimetric charge capacity of silicon, respectively.

Parameter	Description	Value	Taken from
$I(t)$	electric / ionic current	mA	measured by the battery charger
m_A	active mass	175 µg	electrode weight and composition
C_{Si}	silicon gravimetric charge capacity	3578 mAh/g	theoretical gravimetric capacity of silicon ($Li_{15}Si_4$), see main text
A	geometric surface area of electrode	$1.767\ \mathrm{mm}^2$	size of electrode puncher
$d(t)$	electrode thickness or position of separator - electrode interface relative to position of electrode - current collector interface	µm	tomographic data
ϵ	porosity	0.54	composition and volume of the pristine electrode (see main text)
τ	tortuosity	2.5	variable input
k	exchange rate constant of lithium ion transfer from electrolyte to silicon particles	$30\ \mathrm{h}^{-1}$ if $c_{Si} < c_{max}$<br>$0\ \mathrm{h}^{-1}$ else	variable input
D	diffusivity of lithium ions in electrolyte	$1.5 \times 10^{-6}\ \mathrm{cm}^2\mathrm{s}^{-1}$	typical value for carbonate electrolytes at room temperature (see main text)
c_{ini}	initial concentration of lithium ions in the electrolyte	$1\ \mathrm{l}^{-1}\mathrm{mol}$	given by electrolyte manufacturer
dt	grid spacing in time domain	0.2 s	defined to reach convergence of the equations
dx	grid spacing in space domain	2 µm	defined to reach convergence of the equations

Table S1 | Simulation input parameters List of parameters and their values used as input to the simulation presented in the main text.

Table S1 summarizes the different input parameters and their values used for the simulation presented in the main text. Since the tortuosity τ and the rate constant k are unknown, we have varied these input parameters to qualitatively fit the resulting state of charge distribution shown in **Figure 5a** to the experimental results shown in **Figures 3b-c** and **Figure 4f** (see main text).

We also analyse the sensitivity of the simulation results with respect to the input parameters k and τ and the specific charge capacity C of the active material.

Decreasing the rate constant k (**Figure S6a**) or the tortuosity τ (**Figure S6 c**) has the effect of decreasing the ratio T_E/T_S. In this case, the electrode lithiates more homogeneously and the spatial state of charge gradients in the electrode at a given time are less pronounced. However, in the scenario depicted in **Figure S6a**, the given experimental influx of lithium ions causes concentrations in the electrolyte to exceed the initial concentration, which is unrealistic during a potentiostatic lithiation process. In the scenario corresponding to **Figure S6c**, the resulting state of charge gradients are less pronounced than what is experimentally observed.

Increasing the rate constant k (**Figure S6b**) or the tortuosity τ (**Figure S6d**) increases the ratio T_E/T_S, which results in more pronounced state of charge gradients. However, while in

Figure S6 b the gradients are more expressed than what is experimentally observed, the scenario depicted in **Figure S6 d** is again unrealistic because of concentrations in the electrolyte exceeding the initial concentration.

This sensitivity analysis shows that all simulations where (τ, k) parameters deviate from the combination $(\tau = 2.5, k = 30\ \mathrm{h}^{-1})$ used for the simulation in the main text either result in non-physical scenarios or fail to match the experimental data. This implies that (i) the model is sensitive towards variations in τ or k and (ii) that $(\tau = 2.5, k = 30\ \mathrm{h}^{-1})$ is the best fit for the experimental data.

In **Figure S6c**, we show the simulated state of charge distribution, if graphite ($C = 372\ \mathrm{mAh/g}$) not silicon ($C = 3578\ \mathrm{mAh/g}$) were the active material. In this case, the electrode would have been lithiated within approximately $25\ \mathrm{min}$ instead of the $800\ \mathrm{min}$ that are required for the silicon electrode. This illustrates that inhomogeneous state of charge distributions are much more likely to occur in electrodes with a high specific charge capacity.

Finally, we list some of the assumptions implicitly contained in the model:

(i) Charge balance is fulfilled (i.e. lithium ions are counterbalanced by an appropriate amount of electrons in the active material and negatively charged ions in the electrolyte)

(ii) Electrode properties are homogeneous (i.e. an effective medium approach)

(iii) Rate at which lithium ions transfer from the electrolyte to the active material is proportional to the ion concentration in the electrolyte (see transfer term $-k \cdot c_{EL}(z,t)$)

(iv) Secondary effects such as stress-potential coupling in silicon are negligible

Despite these assumptions, the model reproduces the most significant features of the experimental data and allows us to understand the lithiation process on a qualitative level.

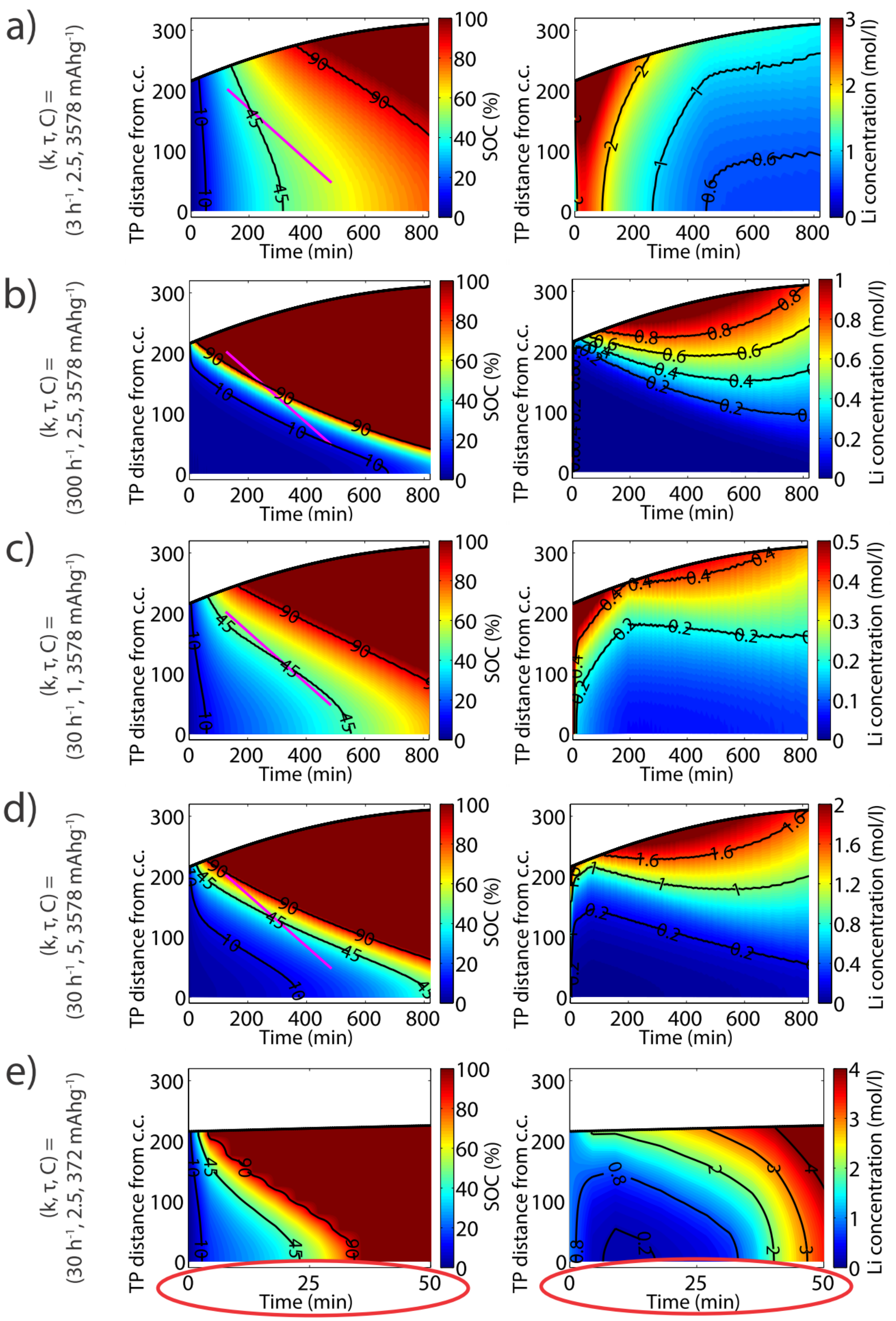

Figure S6 | Sensitivity of the model with respect to *k*, *τ*, and the specific charge capacity C (**a**)-(**e**) Simulation results showing the state of charge distribution in the active material (left column) and the lithium ion concentration distribution in the electrolyte (right column). Input parameters are as defined in **Table S1**, except those specified at the left of the corresponding graphs. In the state of charge maps, the purple line represents the lithiation front discussed in the main text.

Dimensions of the operando cell

Figure S7 depicts the details and dimensions of the operando cell. The cell housing is made from polyether ether ketone (PEEK).

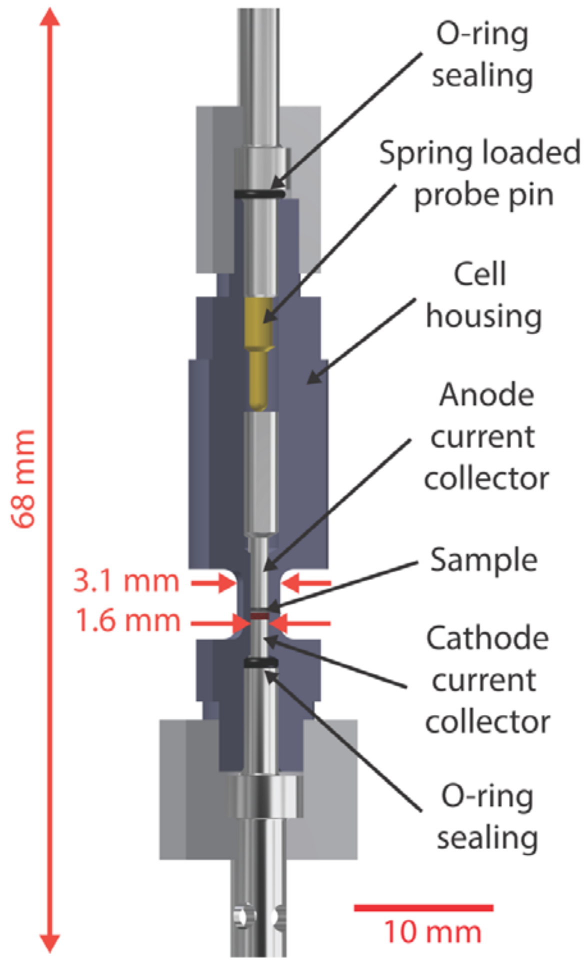

Figure S7 | Dimensions and details of the operando cell Components of the cell are labelled in black, dimensions are labelled in red.

Silicon particle fracture

Figure S8 shows the formation of cracks within a silicon particle upon lithiation.

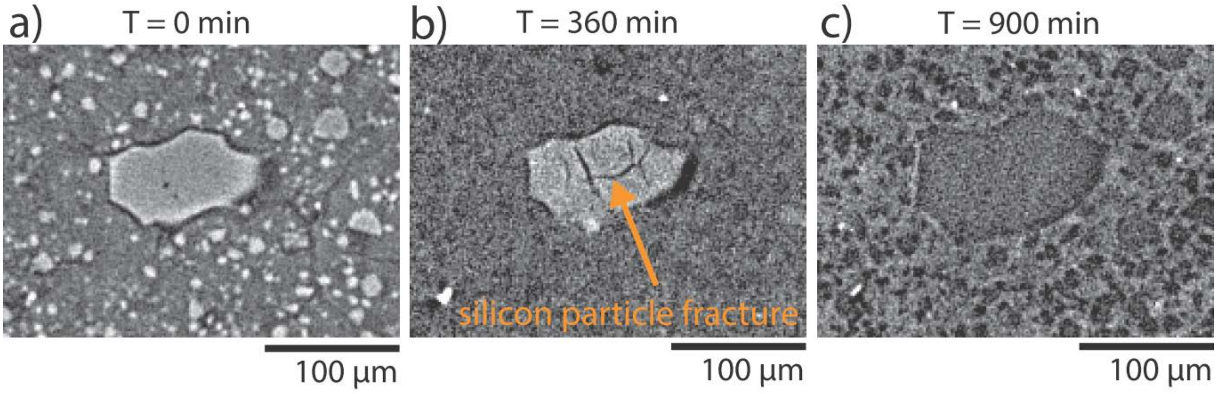

Figure S8 | Silicon particle fracture Corresponding slices through the tomographic data, showing a big silicon particle (**a**) at the onset of lithiation, (**b**) after 360 min, and (**c**) at the end of the experiment.
